# Secondary Metabolites with Antifungal Activities from Mangrove Derived Fungus *Monascus purpureus* WMD2424

**DOI:** 10.3390/md21040200

**Published:** 2023-03-24

**Authors:** Ming-Der Wu, Jih-Jung Chen, Ming-Jen Cheng

**Affiliations:** 1 Bioresource Collection and Research Center (BCRC), Food Industry Research and Development Institute (FIRDI), Hsinchu 300, Taiwan; 2 Department of Pharmacy, School of Pharmaceutical Sciences, National Yang Ming Chiao Tung University (NYCU), Taipei 112, Taiwan; 3 Department of Medical Research, China Medical University Hospital, China Medical University, Taichung 404, Taiwan; 4 Department of Life Science, Fu Jen Catholic University, New Taipei City 242, Taiwan

**Keywords:** *Monascus purpureus* wmd2424, Monascaceae, isoquinoline, antifungal activities

## Abstract

The mold *Monascus*, also called red yeast rice, anka, or koji, has been used as the natural food coloring agent and food additives for more than 1000 years in Asian countries. It has also been used in Chinese herbology and traditional Chinese medicine due to its easing digestion and antiseptic effects. However, under different culture conditions, the ingredients in *Monascus*-fermented products may be changed. Therefore, an in-depth understanding of the ingredients, as well as the bioactivities of *Monascus*-derived natural products, is important. Here, through the thorough investigation into the chemical constituents of *M. purpureus* wmd2424, five previously undescribed compounds, monascuspurins A–E (**1**–**5**), were isolated from the EtOAc extract of mangrove-derived fungus *Monascus purpureus* wmd2424 cultured in RGY medium. All the constituents were confirmed via HRESIMS and 1D- and 2D-NMR spectroscopy. Their antifungal activity was also evaluated. Our results showed that four constituents (compounds **3**–**5**) possessed mild antifungal activity against *Aspergillus niger*, *Penicillium italicum*, *Candida albicans*, and *Saccharomyces cerevisiae*. It is worth mentioning that the chemical composition of the type strain *Monascus purpureus* wmd2424 has never been studied.

## 1. Introduction

Throughout human history, food has been used to satisfy hunger and provide nutrition. Nowadays, food can be widely used not only to eliminate diseases, but also to improve the quality of life. Finding beneficial food resources from the wisdom of your ancestors is a fairly effective strategy. 

Despite plants, fungi (e.g., *Actinomucor* spp., *Amylomyces* spp., *Rhizopus* spp., *Monascus* spp., *Neurospora* spp., *Aspergillus* spp., *Penicillium* spp., *Torulopsis* spp., *Trichosporon* spp., and *Zygosaccharomyces* spp.) also take an important place in producing various food products in fermented forms [1]. Fungi of the genus *Monascus* (Monascaceae) have been used to ferment rice in Asia for centuries. It has been widely utilized as food additives, natural food coloring agent, food antiseptic, and healthy food for nearly two thousand years [2,3]. The production of red yeast rice was used as a Chinese folk medicine, recorded in old Chinese literature as a means of easing digestion and soothing pain. *Monascus* first became known in the West back in 1884, when van Tieghem introduced the usage of red powder (*Monascus ruber*) in Java local populations. Until 1979, Endo et al. isolated monacolin K analogues from *M. ruber* and opened up the investigation of ingredients and bioactivities from *Monascus* [4]. Monacolin K is the same compound as cholesterol-lowering medicine lovastatin, which has been approved by the FDA to become the first commercial statin in 1987 [5].

**Figure 1:**
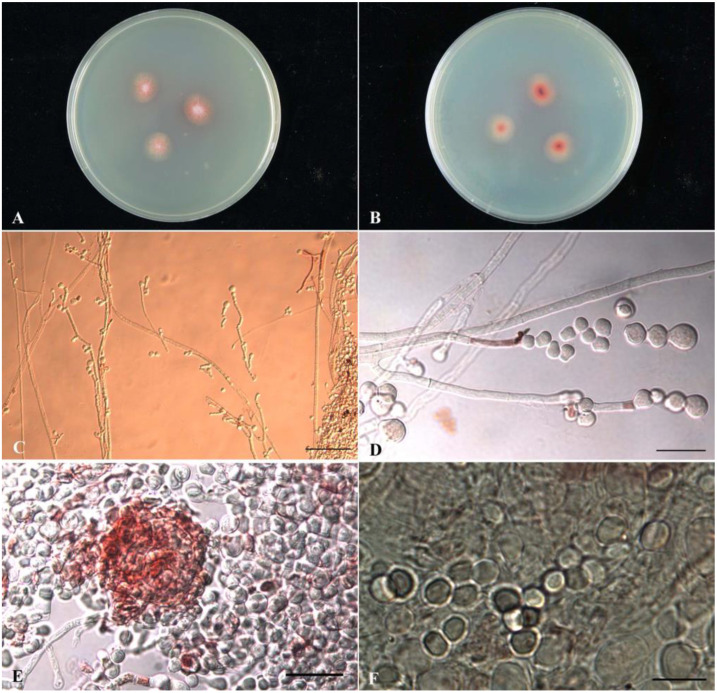
(**A**,**B**) Colony morphology, CYA, 25 °C, cultured for 7 days, (**A**) the front of the colony; (**B**) the back of the colony. (**C**–**F**) Microstructure: (**C**) hyphae and branches (bar = 100 μm); (**D**) conidiophores and conidia (bar = 25 μm); (**E**) ascocarp (bar = 25 μm); (**F**) ascospores (bar = 10 μm).

*Monascus*-fermented rice, also called anka, koji, or red yeast rice, is obtained via the fermentation of rice with fungi of the genus *Monascus*, mainly *M. purpureus, M. pilosus, M. ruber, M. kaoliang,* and *M. anka* [3]. Recently, *Monascus*-fermented rice has been reported for various biological functions. For example, they are helpful for metabolism-related disease with cholesterol-lowering effects [6], cardiovascular diseases [6], and diabetes [7,8]. Also, much evidence has also depicted their anti-inflammation activity [9,10,11], which is also highly associated with cardiovascular disease [12], cancer [13,14,15,16,17], diabetes [18,19], and Alzheimer’s disease [20,21]. Some investigations reveal the anti-microorganism activity of red yeast rice such as anti-bacteria [22,23,24,25,26,27,28,29] and anti-HCV [30]. In recent years, the phytochemical investigation of *Monascus* species have has resulted in the isolation and identification of azaphilones (yellow, orange, and red pigments), monacolins, flavonoids, fatty acids, organic acids, dimerumic acid, and γ-aminobutyric acid, etc. [9,14,27,31,32,33,34]. However, studies on the secondary metabolites of *Monascus* grown in fermentation conditions other than red yeast rice are limited. We recently isolated an unpublished novel strain, named WMD2424, from the mangrove wetland in Chiayi County, which had a unique morphology and possessed antimicrobial activities as determined by our preliminary screening. This strain was determined to be *Monascus purpureus* based on its phenotypic and genotypic data (Figure 1). 

**Figure 2:**
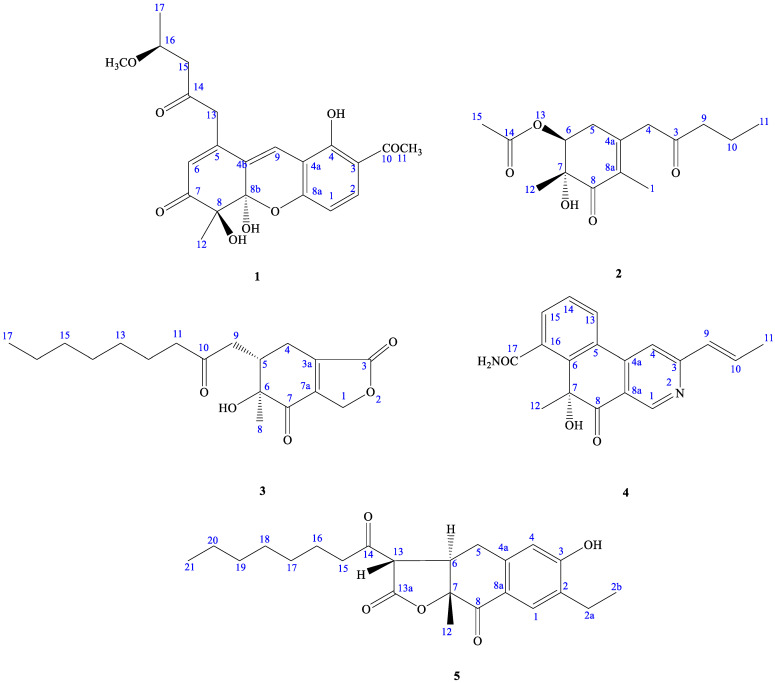
Compounds **1**–**5**, isolated from *Monascus purpureus* wmd2424.

As part of our continuing efforts to explore the chemical diversity of marine fungal metabolites, *Monascus purpureus* WMD2424, fermented using RGY medium (3% rice starch, 7% glycerol, 1.5% polypeptone, 3% soybean powder, 0.2% MgSO_4_, and 0.2% NaNO_3_), was investigated. The scaled-up fermentation and extensive chromatographic separation of the EtOAc extract resulted in the isolation of 5 new metabolites, monascuspurins A–E (**1**–**5**), and their antifungal activity was also evaluated. Herein, we report the structural determination of the new compounds (Figure 2) and the bioactivities of these compounds. 

## 2. Results

### 2.1. Taxonomic Identification (Phenotypic and Genotypic Data) of Monascus purpureus wmd2424

The sample WMD2424 is a filamentous fungal strain collected from the Chiayi mangrove wetland, inoculated in CYA medium, and cultured at 25 °C for 7 days. The diameter of the colony on the CYA plate is 15 mm, and the front color of the colony is reddish orange; the colony is velutinous, without radial grooves (sulcate), exudate (exudate), and soluble pigment (soluble pigment); the back of the colony is reddish orange. Observed under an optical microscope, the mycelium has a septate, and the thin wall is colorless; the conidia are colorless, and the wall is smooth; conidia (conidia) grow on the top or lateral hyphae, with several clusters, pear-shaped, and a truncated bottom, 8.1–16.7 × 6.3–15.5 μm in size, with smooth walls that are colorless; the outer walls of the ascomata are light red but all immature; the same condition was found after 14 days of culture, and only one ascoma was found. The fruit contains ascospores, and the ascospores have a smooth, colorless outer wall and a broad oval shape, with a size of 4.3–5.6 × 3.8–4.8 μm. Partial sequence analysis of the β-tubulin gene fragment was carried out. The total length of the sequence was 1019 bp, compared with the GenBank database, and analyzed and judged with reference to the taxonomic literature. The results showed that the sequence similarity with Monascus purpureus wmd2424 was 99.88% (838/839). The strain was identified as Monascus purpureus according to the colony culture morphology, microstructural characteristics and partial sequence analysis of β-tubulin gene fragment. 

### 2.2. Structure Elucidation of Compounds

**Figure 3:**
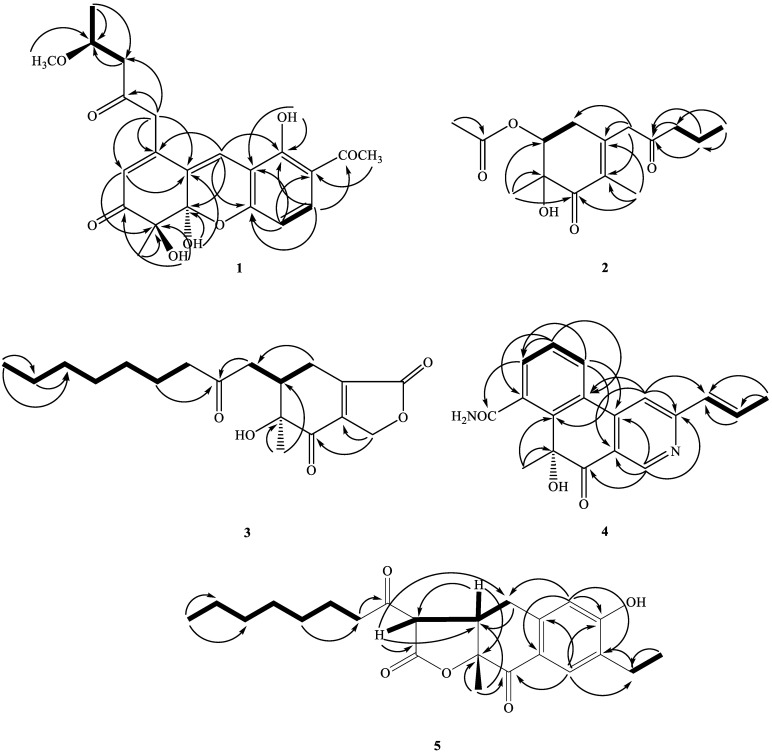
Key COSY (^1^H–^1^H) and HMBC (^1^H→^13^C) correlations of compounds **1**–**5**.

**Figure 4:**
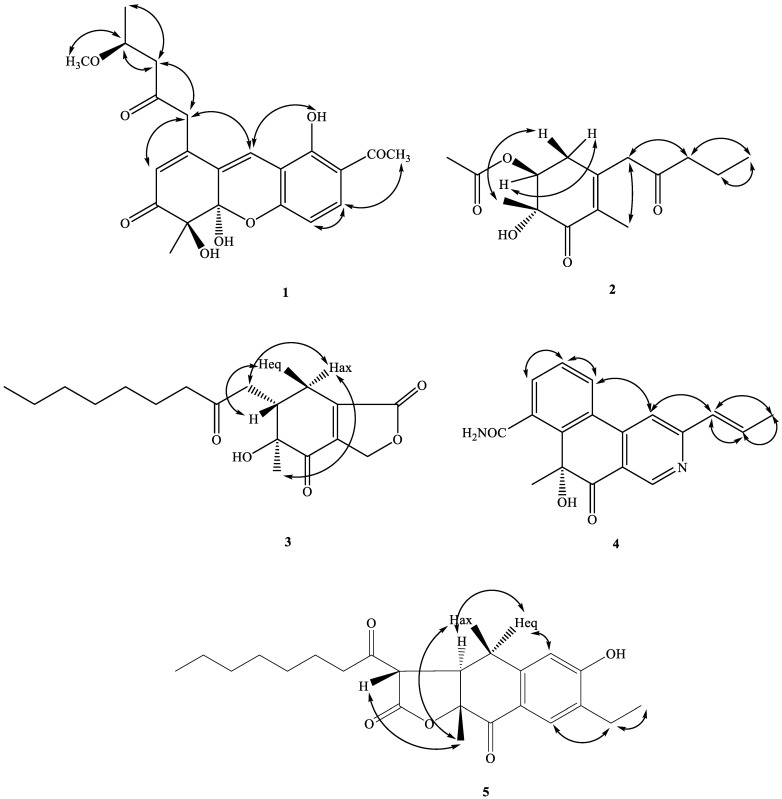
Key NOESY correlations (↔) of compounds **1**–**5**.

**Figure 5:**
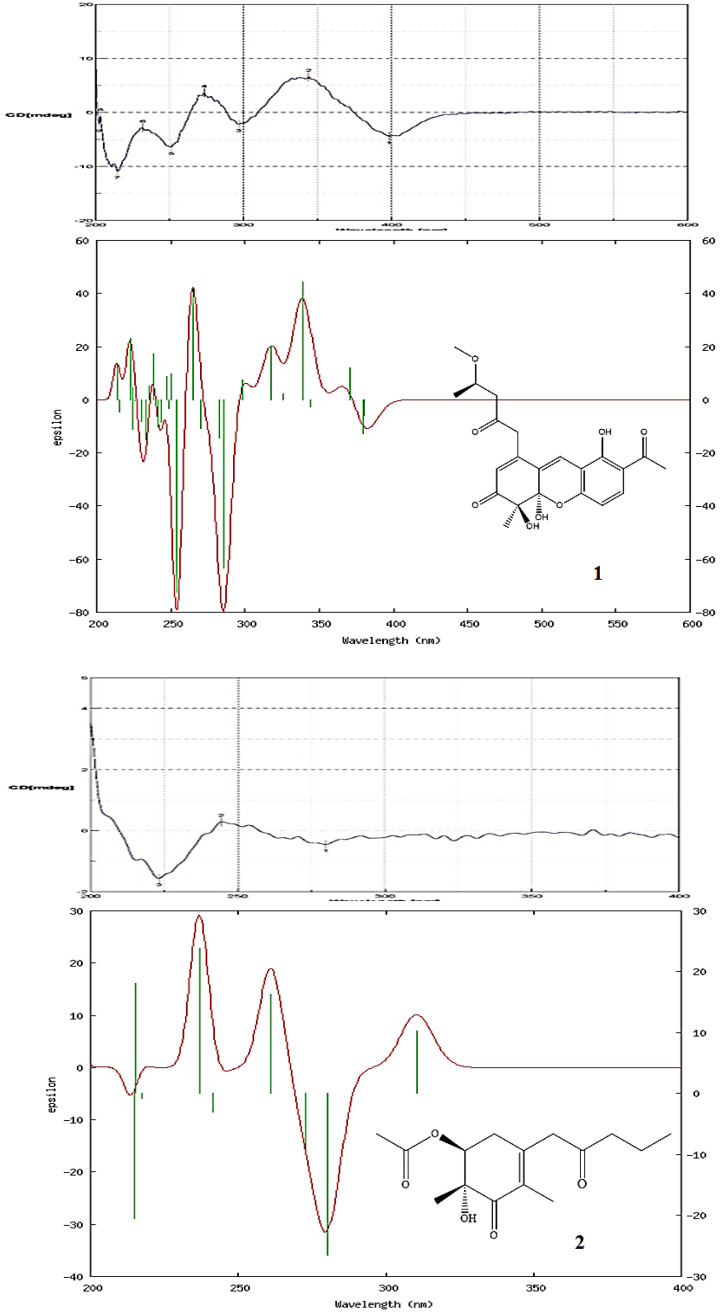

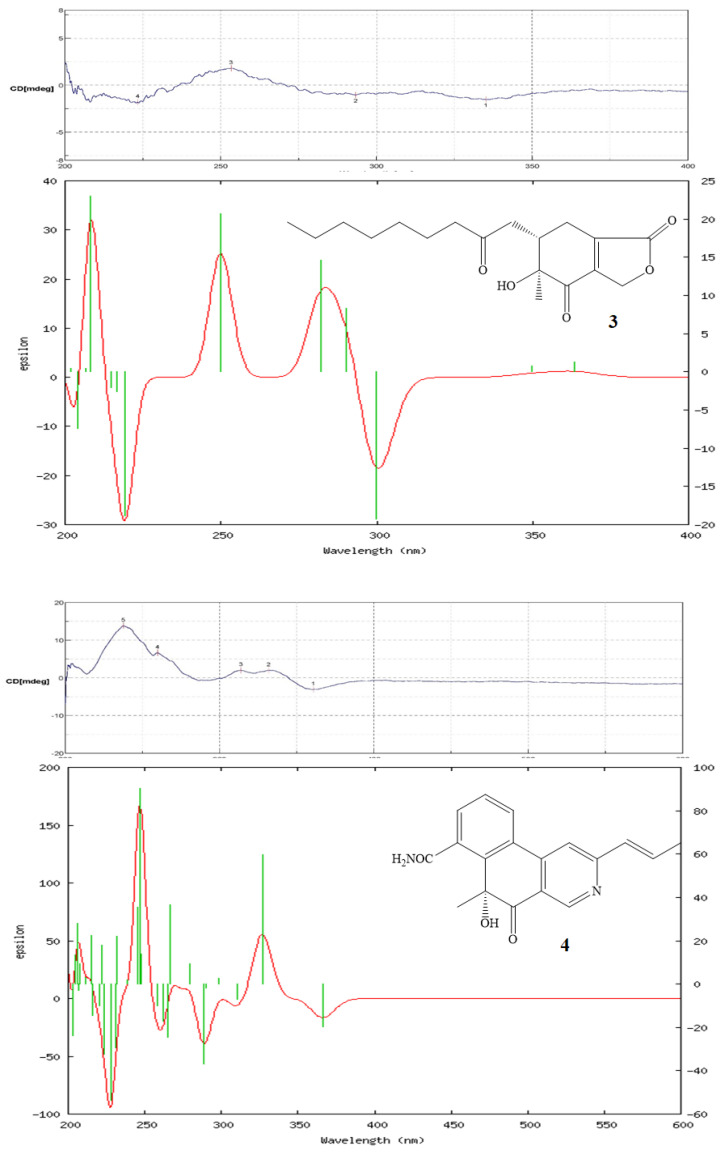

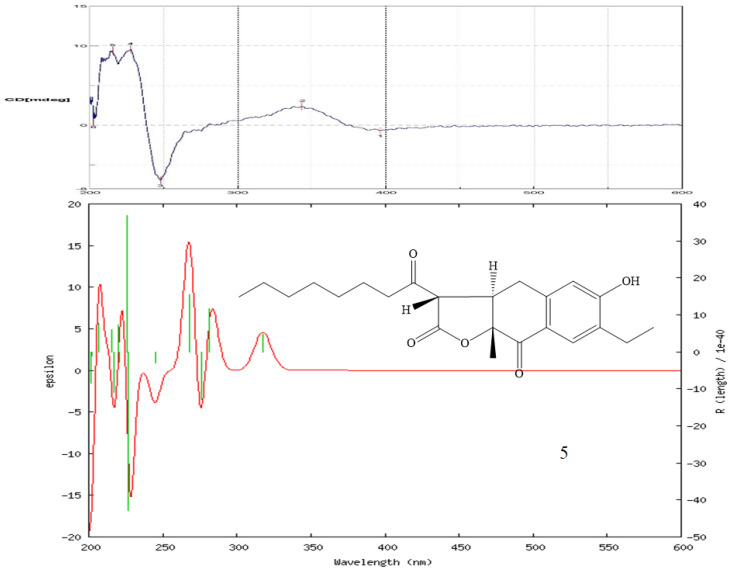
Experimental CD spectra (upper) and the calculated ECD (lower) spectra of compounds **1**–**5**.

Compound **1** was obtained as oil with an [α]^26^_D_: +34.2 (*c* 0.01, CHCl_3_). The molecular formula was established as C_22_H_24_O_8_ via HRESIMS, indicating 11 degrees of unsaturation. The UV spectrum showed maximum absorption at 268 and 360 nm. The IR spectrum showed absorptions at 3406, 1710, and 1680 cm^−1^, corresponding to the hydroxyl group and carbonyl groups. The ^1^H NMR spectroscopic data (Table 1) of **1** show three methyl groups, including one singlet at *δ*_H_ 1.48 (3H, s, H-12), one triplet at *δ*_H_ 1.28 (3H, t, *J* = 6.4 Hz, H-17), and one acetyl at *δ*_H_ 2.60 (3H, s, H-11). It also showed two methylene groups [*δ*_H_ 2.72 (1H, d, *J* = 16.2 Hz, H-15), 2.75 (1H, d, *J* = 16.2 Hz, H-15), 3.72 (1H, d-like, *J* = 17.0 Hz, CH_2_-13), 3.77 (1H, d-like, *J* = 17.0 Hz, CH_2_-13)], one oxymethine [*δ*_H_ 4.25 (1H, m, H-16)], one *meta*-coupling aromatic ring at *δ*_H_ 6.68 (1H, dd, *J* = 8.8 Hz, H-1) and 7.71 (1H, d, *J* = 8.8 Hz, H-2), two olefinic protons at *δ*_H_ 5.94 (1H, s, H-6) and 7.53 (1H, s, H-9), one intramolecular hydrogen bond at *δ*_H_ 13.4 (1H, s, OH-4), and two hydroxyl groups at *δ*_H_ 3.50 (1H, s, OH-8 or OH-8b) and 4.15 (1H, s, OH-8b or OH-8). The ^1^H (Table 1), 2D-NMR (Figure 3 and Figure 4), IR, and UV spectra showed that compound **1** was a xanthene derivative similar to xanthonoides as monasxanthone A [35]. The molecular weight of **1** is 30 units more than **1** and showed another proton peak at *δ*_H_ 4.25 (1H, m, H-16) and 3.21 (3H, s, OCH_3_-16), suggesting the existence of a methoxy group in **1**. The NMR spectra of **1** represent a 4-methoxy-2-oxopentyl moiety at C-5 position in **1** instead of a pentan-2-one in monasxanthone A. Thus, the structure of **1** was elucidated as 7-acetyl-4,8-dihydroxy-1-(4-methoxy-2-oxopentyl)-4-methyl-4,4a-dihydro-3*H*-xanthen-3-one and named monascuspurin A. The relative configuration of **1** was deduced from the NOESY spectrum (Figure 4). The absolute configuration of **1** was further established as (8*S*,8b*R*,16*S*), for the experimental electronic circular dichroism (ECD) curve was in line with its theoretical curve, which was calculated by using the time-dependent density functional theory (TD-DFT) approach [36] (Figure 5). 

Compound **2** was obtained as an optically active colorless oil. [α]^26^_D_: +54.2 (*c* 0.01, CHCl_3_). The molecular formula was determined as C_15_H_22_O_5_ (five degrees of unsaturation) via HR-ESI-MS (*m*/*z* 305.13598, ([M+Na]^+^, C_21_H_30_${\mathrm{NaO}}_{5}^{+}$; calcd. 305.13592)), which was in agreement with the ^1^H- and ^13^C-NMR data (Table 1). The UV spectrum absorption λ_max_ (MeOH) at 242 nm, and a strong IR absorption at 1675 cm^−1^, as well as the observation of the featuring carbon resonances [δ_C_ 132.1 (C-8a), 146.2 (C-4a), and 195.1 (C-8)] in the ^13^C-NMR spectrum (Table 1), revealed the presence of an α,β-unsaturated carbonyl functionality in **1**. The remaining IR spectrum revealed the presence of the OH group (3410 cm^−1^), and ester (1715 cm^−1^), respectively. The ^1^H-NMR spectrum of **2** exhibited signals attributed to one allylic Me (δ_H_ 1.76 (3H, q, *J* = 1.2 Hz, Me-1), signals of *α*-methylene protons of one ketone [δ_H_ 3.33/3.48 (each 1H, d, J = 16.8 Hz, CH_2_-4), 2.45 (2H, t, *J* = 7.8 Hz, CH_2_-9)], one *β*-methylene signals of ketone (δ_H_ 1.61 (2H, sextet, *J* = 7.8 Hz, CH_2_-10)), one aliphatic CH_2_ proton (δ_H_ 2.49 (1H, ddd, J = 18.0, 10.7, 1.2 Hz, Hax-5) and 2.53 (1H, ddd, J = 18.0, 6.2, 1.2 Hz, Heq-5)]), one oxymethine [*δ*_H_ 4.83 (1H, dd, *J* = 10.7, 6.2 Hz, H-6)], one acetoxy group [*δ*_H_ 2.09 (3H, s, H-15)], and one terminal Me moiety (*δ*_H_ 0.92 (3H, t, *J* = 7.8 Hz, H-11)). Fifteen C-atom signals (Table 2) corresponding to six quaternary C-atoms (including three carbonyl groups, one oxygenated quaternary carbon), one OCH, two olefinic carbons, four CH_2_, and four CH_3_ groups were observed in the ^13^C-NMR and DEPT spectra. 

The ^1^H- and ^13^C-NMR spectra of **2** (Table 1 and Table 2) were similar to those of monaphilone C [31], except that an acetoxy moiety at C-6 of **2** replaced a 2-oxoheptyl moiety at C-6 of monaphilone C. The planner structure of **2** was confirmed using the COSY and HMBC experiments (Figure 3). The stereochemistry of **2** was proposed on the basis of the NOESY experiments (Figure 4). The H-6/CH_3_-12 has no correlation in the NOESY spectrum (Figure 4) represented acetoxy group and CH_3_-12 are *syn*-form. The physicochemical data and NMR spectra of compound **2** and the known compound monapurpureusone [31] are similar; the only difference is that the specific rotation of monapurpureusone is negative, while the specific rotation of **2** is positive, and it can be inferred that the two are stereoisomers. On comparing the reference to the (6*S*,7*R*)-configuration of FK17-P2b (α]^26^_D_: + 26.0 (c 0.1, MOH)) [37], the relative configuration of **2** can be assigned as *rel*-(6*S*,7*R*)-configuration, and named monascuspurin B. The absolute configuration of **2** was defined via a comparison of the experimental and calculated ECD data (Figure 5). Therefore, the absolute configuration of **2** was undoubtedly determined as (6*S*,7*R*). 

Compound **3** was isolated as oil with [α]^26^_D_: +74.2 (*c* 0.01, CHCl_3_). Its molecular formula was determined to be C_18_H_26_O_5_ based on the HRESIMS [M+Na]^+^ peak at 345.16780 (Calcd.: C_18_H_26_O_5_, 345.16779), referring six degrees of unsaturation. The maximum absorption of an IR spectrum showed the presence of the hydroxyl group (3410 cm^−1^), γ-lactone (1770 cm^−1^), and α,β-unsaturated C=O (1715 cm^−1^). The UV spectrum exhibited the absorption band at 285 nm. The CD spectrum showed a positive Cotten effect at 250 nm and depict the negative Cotten effect at 225, 290, and 335 nm. 

The ^1^H-NMR spectrum (Table 1) displayed an oxononyl group at [*δ*_H_ 0.90 (3H, t, *J* = 7.2 Hz, H-17), 1.20–1.35 (8H, m, H-13~H-16), 1.55–1.60 (2H, m, H-12), 2.44–2.46 (2H, m, H-11)], one methyl group [*δ*_H_ 1.24 (3H, s, H-8)], signals of the *α*-methylene protons of one ketone [*δ*_H_ 2.49–2.52 (1H, m, 1H of CH_2_-9), 3.03 (1H, dd, J = 18.0, 3.2 Hz, 1H of CH_2_-9), and 2.44–2.47 (2H, m, CH_2_-11)], one oxymethylene [*δ*_H_ 4.89 (1H, dd, J = 18.0, 4.5 Hz, 1H of CH_2_-1), 5.05 (1H, dd, J = 18.0, 3.3 Hz, 1H of CH_2_-1)], one non-equivalent methylene proton at [*δ*_H_ 2.10–2.12 (1H, m, 1 H of CH_2_-4), 2.95 (1H, ddd, J = 19.0, 4.5, 3.3 Hz, 1 H of CH_2_-4)], and one methine [*δ*_H_ 2.80–2.82 (1H, m, H-5)]. Eighteen C-atom signals (Table 2) corresponding to six quaternary C-atoms, one CH, nine CH_2_, and two CH_3_ groups, were observed in the ^13^C-NMR and DEPT spectra. Since four out of six unsaturation equivalents were accounted for via the above-mentioned ^13^C-NMR data, **1** was inferred to have two rings (one as a six-membered and another as a five-membered ring). In addition, two rings were further determined as a cyclohex-2-enone skeleton combined with one *γ*-lactone ring via the detail HMBC and COSY analyses. 

The ^1^H- and ^13^C-NMR spectra of **3** (Table 1) were similar to those of monaphilone A [31]; the major difference was the presence of signals for an *γ*-lactone attached to C-3a and 7a in **3**, instead of signals for a 4*H*-pyran group in monaphilone A [31]. HMBC correlations between the H-atom signals at δ_H_ 4.89/5.05 (CH_2_(1)) and the C-atom signals at δ_C_ 198.3 (C-7) once indicated that the *γ*-lactone was located at C-3a and 7a of the cyclohex-2-enone ring. The relative configuration of **3** was derived using a NOESY spectrum (Figure 4) and a comparison with similar compounds [31], the relative configuration of which was based on a NOESY analyses. No NOEs for H-5/Me_ax_-8 and H_ax_-4 indicated that Me-8 and H_ax_-4 were on the same side of the molecular plane, tentatively assumed as *α*-orientation. 

The H-5 was occupied at axial *β*-oriented, which was further confirmed by the NOE H-5/H_eq_-4. The relative configuration at C-5 and 6 were determined to be (5S*,6S*) based on the correlation between the [α]_D_ value and the known configuration at C-5/C-6 for monaphilone A type derivatives [31]. In order to determine the absolute configuration of **3**, the theoretical electronic circular dichroism (ECD) spectra of 4 possible stereoisomers were calculated using a time-dependent density-functional theory (TDDFT) calculation, and the calculated ECD curve of (5*S*,6*S*) revealed good agreement with the experimental spectrum of **2** (Figure 5). Therefore, the absolute configuration of **3** was assigned as (5*S*,6*S*) and named as monascuspurin C. 

Compound **4** was obtained as colorless oil. The molecular formula was determined as C_18_H_16_N_2_O_3_ on the basis of the [M+Na]^+^ peak at *m*/*z* 331.10588 (calcd. 331.10586 for C_18_H_16_NaN_2_O_3_) in its HR-ESI-MS. The UV absorptions (λ_max_ 220, 252, and 312 nm) confirmed the presence of a pyridine moiety [38]. IR absorption bands were assigned to amide (3400 cm^−1^), multiple carbonyls C=O (1712 and 1656 cm^−1^), and the pyridine ring (1589, 1535, and 1458 cm^−1^) functional groups. Twelve indices of hydrogen deficiency (IHD) were determined from the molecular formula, ^13^C-NMR (Table 2), and DEPT spectra. The CD spectrum showed positive Cotten effect at 240, 262, 319, and 333 nm, and negative Cotten effect at 365 nm. 

Interpretation of the ^1^H-NMR spectrum of **4** (Table 1) exhibited the signals of one 2,4,5-trisubstituted pyridine ring [δ_H_ 9.03 (1H, *s*, H-1), 7.59 (*s*, H-4)], one trans-propenyl unit [δ_H_ 2.05 (3H, dd, *J* = 6.8, 1.8 Hz, H-11), 6.65 (1H dq, *J* = 15.6, 1.8 Hz, H-9), 7.13 (1H, dq, *J* = 15.6, 6.8 Hz, H-10)], one Me group [δ_H_ 1.85 (3H *s*, Me-12)], as well as one ABC system aromatic ring [*δ*_H_ 7.70 (1H, t, *J* = 8.0 Hz, H-14), 7.90 (1H, dd, *J* = 8.0, 0.8 Hz, H-15), and 8.04 (1H, dd, *J* = 8.0, 0.8 Hz, H-13)]. The ^13^C and DEPT NMR spectra indicated (Table 2) that compound **4** is a pyridine derivative with signals for 18 C-atoms, which were classified as nine quaternary C-atoms comprising six olefinic C-atoms, one amide C-atom (δ_C_ 168.5 (C-17), one ketone groups (δ_C_ 192.8 (C-8)), one oxygenated quaternary carbon [*δ*_C_ 84.9 (C-7)], one Me group (δ_C_ 27.3 (C-12), and one trans-propenyl unit [*δ*_C_ 131.8 (C-9), 137.2 (C-10), 18.8 (C-11)]. 

The ^1^H- and ^13^C-NMR spectra of **4** (Table 1) were similar to those of monascopyridine C and D [38]; the major difference was the presence of signals for ABC system aromatic ring attached between C-5 and C-6 in **4**, instead of signals for an alkyl groups in monascopyridine C and D. HMBC correlations between the H-atom signals at δ_H_ 8.04 (H-13) and the C-atom signals at δ_C_ 151.0 (C-6), and 143.5 (C-4a) and δ_H_ 7.70 (H-14) and the C-atom signals at δ_C_ 126.7 (C-5), indicated that the ABC system aromatic ring was bounded at C-5 and 6. The other key correlations of HMBC were illustrated in Figure 3. 

Furthermore, the attachment of the amide to C-17, the methyl group to C-7, and the trans-propenyl group located at C-3, were disclosed according to the HMBC cross-peaks of *δ*_H_ 7.90 (H-15)/*δ*_C_ 168.5 (C-17), *δ*_H_ 1.85 (H-12) to C-6/C-7/C-8, and *δ*_H_ 7.59 (H-4) to C-9. 

On the basis of the evidence, the entire structure of **4** was confirmed and named monascuspurin D. The relative configuration at C-7 was determined to be 7*R* based on the correlation between the [α]^26^_D_:+ 15.9 (*c* 0.01, CHCl_3_) and the known configuration at C-7 for (*R*)-2-hydroxy-2-methylcyclohexanone derivatives [39]. The absolute configuration of **4** was defined via a comparison of the experimental and calculated ECD data (Figure 5). Therefore, the absolute configuration of **4** was determined as 7*R*. 

Compound **5** was obtained as an optically active oil. [α]^26^_D_: +56.7 (*c* 0.01, CHCl_3_). The molecular formula was determined as C_23_H_30_O_5_ on the basis of the [*M*+H]^+^ peak at *m*/*z* 409.19912 (calcd. 409.19909 for C_23_H_30_NaO_5_) in its HR-ESI-MS. The UV absorptions (λ_max_ 235 and 285 nm) confirmed the presence of a benzenoid nucleus. The bands at 3400, 1780, 1695, and 1615/1577 cm^−1^ in the IR spectrum revealed the presence of a hydroxyl group, γ-lactone, and aromatic ring, respectively. Nine indices of hydrogen deficiency (IHD) were determined from the molecular formula, ^13^C-NMR (Table 1), and DEPT spectra. The ^1^H-NMR and ^13^C-NMR spectra (Table 2) of **5** were similar to those of ankaflavin [9], except that a 2-ethylphenol group of **5** replaced a (*E*)-6-(prop-1-en-1-yl)-2*H*-pyran group at C-4a–C-8a of ankaflavin. Further confirmation using the HMBC correlations (Figure 3) of H-1/C-3, 4a, 2a, H-4/C-2, 3, 5, 8a, and H-2b/C-2, 2a, verified the junction of the 2-ethylphenol unit at C-4a and C-8a. The correlations of H-1/H-2a and H-4/CH_2_-5 were also observed in the NOESY experiment (Figure. 4) and further supported the position of each aromatic substitution. The ^1^H- and ^13^C-NMR, COSY (Figure 3), NOESY (Figure 4), HSQC, and HMBC (Figure 3) experiments confirmed the structure as 7-ethyl-3-hexanoyl-6-hydroxy-9a-methyl-3a,9adihydronaphtho[2,3-*b*]furan-2,9(3*H*,4*H*)-dione, and designated monascuspurin E.

The dextrorotatory optical activity of **5**, gathered from the NOESY spectrum (Figure 4), indicates that Hax-5 is correlated to H-12 and H-13, and H-6 has no NOE contacts with Hax-5, H-12, and H-13. It can be concluded that Hax-5, H-12, and H-13 are on the same side, and H-6, H-12, and H-13 are on the opposite side, and once again it indicated that the relative configuration of **5** is (6*R*,7*R*,13*S*), as in the case of ankaflavin [9]. In order to determine the absolute configuration of **5**, the theoretical ECD spectra of all possible stereoisomers were calculated using the TDDFT calculation, and the calculated ECD curve of the isomer (6*R*,7*R*,13*S*) revealed a good agreement with the experimental one (Figure 5). Therefore, the absolute configuration of **5** was assigned as (6*R*,7*R*,13*S*)-form and named as monascuspurin E.

## 3. Discussion

Red yeast rice has been used in food and traditional Chinese medicine since ancient times. In recent years, research has also found that red yeast rice bacteria can produce many active secondary metabolites. In order to further explore the efficacy of different strains of red yeast rice and expand the application range of red yeast rice, in this study, a strain wmd2424 was isolated from the mangrove forest in Chiayi Wetland, and the strain was identified as *Monascus purpureus* via the results of colony culture morphology, microstructural characteristics, and partial sequence analysis of the β-tubulin gene fragment. After liquid fermentation using RGY medium, extraction with ethyl acetate, and analysis of its metabolites, a total of six new compounds were obtained.

To the best of our knowledge, this is the first report of isoquinoline-type metabolites from the edible fungi genus *Monascus*. These results demonstrate that *Monascus* produces unique and diverse metabolites in different fermentation conditions and soil-derived collections. Therefore, in a special ecological environment, more natural products with biological activity may be found by searching for *Monascus* species.

### Biological Studies

Culture broth from *M*. *purpureus* wmd2424 was tested for antifungal activity against the following fungi: *Aspergillus niger* (BCRC-31512), *Penicillium italicum* (BCRC-30567), *Candida albicans* (BCRC-21538), and *Saccharomyces cerevisiae* (BCRC-20822). The antifungal data are shown in Table 3 and the clinically used antifungal drug ketoconazole was employed as a positive control.

Our results indicate that compounds **3**–**5** have moderate antifungal activity compared to ketoconazole, with **1** being weaker. From the results of the antifungal tests, the following conclusions can be drawn about these isolates: (a) within the novel strain, the 2,3-dimethylcyclohex-2-en-1-one (compound **2**) and γ-lactone (compound **3**) showed antifungal activities with inhibition zones of 29, 28, 27, and 30 mm, and 29, 29, 36, and 21 mm against *Aspergillus niger* (BCRC-31512), *Penicillium italicum* (BCRC-30567), *Candida albicans* (BCRC-21538), and *Saccharomyces cerevisiae* (BCRC-20822), respectively. (b) The xanthonoids (compound **1**) exhibited weak antifungal activities against the *Aspergillus niger* (BCRC-31512), *Penicillium italicum* (BCRC-30567), *Candida albicans* (BCRC-21538), and *Saccharomyces cerevisiae* (BCRC-20822) strains. (c) The other type of isoquinoline, Monascuspurin D (compound **4**), indicated effective inhibition zones of 32, 28, 31, and 28 mm against *Aspergillus niger* (BCRC-31512), *Penicillium italicum* (BCRC-30567), *Candida albicans* (BCRC-21538), and *Saccharomyces cerevisiae* (BCRC-20822), respectively. (d) The azaphilone compound **5** exhibited moderate antifungal activities against the *Aspergillus niger* (BCRC-31512) and *Candida albicans* (BCRC-21538) strains (Table 3).

The inhibitory activity of compounds **3**–**5** against *A*. *niger*, *P*. *italicum*, *C*. *albicans*, and *S*. *cerevisiae* was further tested using the method described in the experimental section (Table 4). Compound **2** has inhibitory activity against *S*. *cerevisiae*, with MIC values of 43.45 μg/mL. Compound **3** has inhibitory activity against *C*. *albicans*, with an MIC value of 32.87 μg/mL. Compound **4** was found to have moderate inhibitory activity against the *A*. *niger*, and *C*. *albican* strains with MIC values ranging from 29.65 and to 58.43 μg/mL. They were less biologically active than the reference compound, ketoconazole, which had MIC values of 4.10, 5.34, 10.88, and 3.57 μg/mL against *A*. *niger*, *P*. *italia*, *C*. *albicans*, and *S*. *cerevisiae*, respectively. In this bioassay, no antifungal activity (MIC > 100) was observed for compound **5** at concentrations below 100 μg/mL.

## 4. Materials and Methods

### 4.1. General Experimental Procedures

For the TLC, we used silica gel 60 F254-precoated plates (Merck); for column chromatography (CC), we used silica gel 60 (70–230 or 230–400 mesh, Merck) and Spherical C18 100A Reversed Phase Silica Gel (RP-18) (particle size: 20–40 μm) (Silicycle). For the HPLC analysis, we used a spherical C18 column (250 mm × 10 mm, 5 μm) (Waters) and LDC-Analytical-III apparatus. For the UV spectra, we used a Jasco UV-240 spectrophotometer, with λmax (log ε) in nm. For optical rotation, we used a Jasco DIP-370 polarimeter, in CHCl3. For the IR spectra, we used a Perkin-Elmer-2000 FT-IR spectrophotometer, with ν in cm^−1^. For the 1H-, 13C-, and 2D-NMR spectra, we used Varian-VNMRS-600 and Varian-Unity-Plus-400 spectrometers; δ in ppm relative to Me4Si, J in Hz. For the ESI and HRESIMS, we used a Bruker APEX-II mass spectrometer, in *m*/*z*.

### 4.2. Microorganism, Cultivation, and Preparation of the Strain

This WMD2424 strain was isolated from the mangrove wetland collected in Chiayi County, Taiwan, using HV agar and cultured at 28 °C for 3 weeks. A voucher specimen was immersed in 15% glycerol–water solution at −80 °C and deposited at the Bioresource Collection and Research Center (BCRC) of the Food Industry Research and Development Institute (FIRDI). Analysis of the ITS rDNA using the BLAST database screening provided a 99.9% match with *Monascus purpureus*, whose sequence has been submitted to GenBank.

To each 500-mL flask containing 150 mL of liquid RGY medium (3% rice starch, 7% glycerol, 1.5% polypeptone, 3% soybean powder, 0.2% MgSO4, and 0.2% NaNO_3_) were added 10 mL of fungal inocula and incubated at 25° for 2 weeks on a rotary shaker at the speed of 100 circles/min without illumination. A total of 14.0 L of fungal fermented broth was harvested and then filtered to remove fungal mycelium.

### 4.3. Isolation and Characterization of Secondary Metabolites

Liquid fermentate of M. purpureus (14.0 L) was extracted with BuOH to yield a BuOH extract (16.9 g), which was partitioned in EtOAc–H_2_O (1:1; 2 L × 3) to produce an EtOAc-soluble fraction (8.9 g) and an H_2_O-soluble fraction. The active EtOAc-soluble fraction (8.9 g) was subjected to silica gel column chromatography (CC) using CH_2_Cl_2_–MeOH (100:1) as the primary eluent, gradually increasing the eluent polarity with MeOH to produce 10 fractions (Frs. 1–Frs. 10). Fr. 2 was subjected to RP-18 silica gel CC using H_2_O–acetone (2:1) as the eluent to produce 5 fractions (Frs. 2-1–2-5), Fr. 2-5 (432 mg) was subjected to silica gel CC using CH_2_Cl_2_–EtOAc (3:1) as the eluent to produce 4 fractions (Frs. 2-5-1–Frs. 2-5-4), Fr. 2-5-3 was further subjected to silica gel CC using CH_2_Cl_2_–EtOAc (2:1) as the eluent to give **1** (1.2 mg) and **2** (3.0 mg). Fr. 3 was subjected to RP-18 silica gel CC using H_2_O–acetone (1:1) as the eluent to obtain 8 fractions (Frs. 3-1–3-8), Fr.3-8 was further subjected to silica gel CC using CH_2_Cl_2_–acetone (1:1) as the eluent to give 11 fractions (Frs. 3-8-1–Frs. 3-8-11), Fr. 3-8-10 was purified with prep. TLC (CH_2_Cl_2_/EtOAc 6:1) to obtain **4** (1.8 mg). Fr. 5 (1132 mg) was subjected to RP-18 silica gel CC using H_2_O–acetone (1:1) as the eluent to give **3** (1.2 mg) and **5** (3.3 mg).

Monascuspurin A (compound **1**): Oil. [α]^26^_D_: +34.2 (*c* 0.01, CHCl_3_). UV (MeOH) λ_max_ (log *ε*) 268 (4.11), 360 (3.89) nm. IR *ν*_max_ (neat) 3406 (OH), 1710, 1680 (C=O), 1615, 1450, 1406 (aromatic ring) cm^−1^. CD (MeOH) λ_ext_ 215 (Δε –10.9), 232 (Δε –4.2), 251 (Δε –7.9), 273 (Δε +5.2), 296 (Δε –2.3), 342 (Δε +7.3), 400 (Δε –6.7) nm. ESI-MS *m*/*z* 439 [M+Na]^+^. ^1^H NMR (600 MHz, CDCl_3_): see Table 1. HRESI-MS *m*/*z*: 439.13640 [M+Na]^+^ (calculated for C_22_H_24_O_8_Na, 439.13636).

Monascuspurin B (compound **2**): Oil. [α]^26^_D_: + 54.2 (*c* 0.01, CHCl_3_). UV (MeOH): 242 (3.98) nm. IR (neat): 3410 (OH), 1715 (C=O), 1675 (C=O) cm^−1^. CD (MeOH) λ_ext_ 225 (Δε –1.9), 241 (Δε +0.9), 282 (Δε –0.3) nm. ^1^H NMR (600 MHz, CDCl_3_): see Table 1; ^13^C NMR (150 MHz, CDCl_3_): see Table 2. ESI-MS *m*/*z* 305 [M+Na]^+^. HRESI-MS *m*/*z*: 305.13598 [M+Na]^+^, (calculated for C_15_H_22_O_5_Na, 305.13592).

Monascuspurin C (compound **3**): Oil. [α]^26^_D_: +74.2 (*c* 0.01, CHCl_3_). UV (MeOH): 285 (3.26) nm. IR (Neat): 3410 (OH), 1770, 1715 (C=O) cm^−1^. CD (MeOH) λ_ext_ (Δε): 225 (Δε –1.89), 250 (Δε +1.79), 290 (Δε –1.08), 335 (Δε –1.69) nm. ^1^H-NMR (600 MHz, CDCl3): see Table 1; ^13^C-NMR (150 MHz, CDCl_3_): see Table 2. ESI-MS *m*/*z* 345 [M+Na]^+^. HRESI-MS *m*/*z*: 345.16780 [M+Na]^+^, C_18_H_26_O_5_ (calculated for C_15_H_13_O, 345.16779.

Monascuspurin D (compound **4**): oil; [α]^26^_D_: + 15.9 (*c* 0.01, CHCl_3_); UV (MeOH): 220 (4.01), 252 (4.22), 312 (3.89) nm; IR (neat): 3400 (OH), 1712, 1656 (C=O), 1589, 1535, 1458 (pyridine) cm^−1^; CD (MeOH) λ_ext_ (Δε) 240 (Δε +13.19), 262 (Δε +5.13), 319 (Δε +1.98), 333 (Δε +2.01), 365 (Δε −2.81) nm. ^1^H-NMR (600 MHz, CDCl_3_): see Table 1; ^13^CNMR (150 MHz, CDCl_3_): see Table 2; ESI-MS *m*/*z* 331 [M+Na]^+^; HRESIMS *m*/*z* 331.10588 [M+Na]^+^ (calculated for C_18_H_16_NO_4_, 331.10586).

Monascuspurin E (compound **5**): oil; [α]^26^_D_: +56.7 (*c* 0.01, CHCl_3_); UV (MeOH): 235 (4.22), 285 (3.89) nm; IR (neat): 3400 (OH), 1780, 1695 (C=O), 1615, 1577 (aromatic ring) cm^−1^; ^1^H-NMR (600 MHz, CDCl_3_): see Table 1; ^13^C-NMR (150 MHz, CDCl_3_): see Table 2; ESI-MS *m*/*z* 409 [M+Na]^+^; HRESIMS *m*/*z* 409.19912 [M+Na]^+^ (calculated for C_23_H_30_O_5_Na, 409.19909).

#### Computational Methods

The theoretical ECD curves of compounds **1**–**5** were calculated by using Gaussian 09, Revsion E.01. software. Conformational searches were performed using Spartan’14 software with the Molecular Merck force field (MMFF). ECD spectra of conformers with a Boltzmann distribution over 2% were calculated via the TD-DFT method at the B3LYP/6.311+G (d,p) level in MeOH. According to a Gaussian band shape with a 0.2 eV exponential half-width from the dipole-length dipolar and rotational strengths, the theoretical ECD spectra were generated using the SpecDis 3.0.

### 4.4. Antifungal Activity Assays

The assays tested for the presence of microorganisms. The in vitro antifungal activity of compounds **1**–**5** was tested against a panel of laboratory control strains belonging to the Bioresource Collection and Research Center (BCRC) in Hsinchu, Taiwan, namely, the fungal organisms *Aspergillus niger* (BCRC-31512), *Penicillium italicum* (BCRC-30567), *Candida albicans* (BCRC-21538), and *Saccharomyces cerevisiae* (BCRC-20822).

#### 4.4.1. Via Disk Diffusion Assay

Antifungal susceptibility testing of the isolated compounds was performed with the following strains: *Aspergillus niger*, *Penicillium italicum*, *Candida albicans*, and *Saccharomyces cerevisiae* using the disk diffusion method and the following CLSI guidelines were applied: M44-A and M44-S2 for yeasts [40,41] and M-51P for filamentous fungi. A standard disk of ketoconazole was used as a positive control, while a disk imbued with 50 μL of pure DMSO was used as a negative control. The diameters of the inhibition zones were measured in millimeters by means of a slide caliper. Each test was performed in triplicate, and the results were analyzed for statistical significance [40,41,42].

#### 4.4.2. Via Broth Dilution Assay

The MIC determination for the antifungal assay was performed according to the Clinical and Laboratory Standard Institute (CLSI) using the broth dilution assay method [43,44,45]. Extract stock solutions and partitions were prepared in 5% DMSO, and twofold serial dilutions were prepared in RPMI in 96-well microtiter plates (Corning Incorporated, Corning, NY, USA). The final concentrations ranged from 0.98 to 2.000 g mL^−1^. Test organisms (100 μL) were added to each well in microtiter plates. The growth control contained medium and inoculum. Blank controls contained medium only. The microtiter plates were then incubated at 35 °C and the endpoints were read after 48 h. The lowest concentration for each test compound at which color change occurred was recorded as its primary MIC value. The average of primary values from three individual tests were calculated, and the average was taken as the final MIC value for each of the test compounds.

## 5. Conclusions

Red yeast rice is a well-known material which has been widely used for decades, but the chemistry and bioactivity of the constituents are still not so clear. Previous investigation of *Monascus* species had isolated different skeleton constituents, mainly azaphilones and monacolin analogs. However, some minor compounds such as benzenoid derivatives or other types of compounds from *Monascus* species have received less attention. Accordingly, it is still worth investigating the ingredients and bioactivity of red yeast rice.

In this report, we committed to explore unusual skeleton compounds in *M. purpureus* wmd2424, and successfully found new xanthonoid, cyclohexenone, γ-lactone, isoquinoline, and azaphilone skeleton compounds. Xanthonoids are yellow pigments in a C_6_-C_1_-C_6_ system and restricted in a few families of higher plants, some fungi and lichens, and has seldom been found in *Monascus* spp. [46] This is the second report of isolating xanthonoids from *Monascus spp*, which represent different yellow azaphilone pigments (monascin, ankaflavin) from this genus. The structures of these isolates were determined using spectroscopic experiments. The BuOH soluble fraction from the *M. purpureus* wmd2424 fermentation broth was tested for antifungal activities. Our results indicated that compounds **3**–**5** displayed moderate antifungal activities against *Aspergillus niger*, *Penicillium italicum*, *Candida albicans*, and *Saccharomyces cerevisiae*. It is worth mentioning that the chemical composition of *M. purpureus* wmd2424 has never been studied. The result indicated *M. purpureus* wmd2424 could produce more metabolites with extensive antifungal activity, and that its metabolites in other mediums were worth being studied further.

## Figures and Tables

**Table 1:** ^1^H-NMR data for Compounds **1**–**5** in CDCl_3_ (*δ* in ppm, *J* in Hz, and 600 MHz in CDCl_3_).

No.	1	2	3	4	5
1	6.68 (1H, dd, *J* = 8.8)	1.76 (3H, q, *J* = 1.2)	4.89 (1H, dd, *J* = 18.0, 4.5) 5.05 (1H, dd, *J* =18.0, 3.3)	9.05 (1H, s)	7.91 (1H, s)
2	7.71 (1H, d, *J* = 8.8)				
2a					2.64 (3H, q, *J* = 7.2)
2b					1.26 (3H, *J* = 7.2)
4		3.33 (1H, d, *J* = 16.8) 3.48 (1H, d, *J* = 16.8)	2.10–2.12 (1H, m) 2.95, d (1H, dd, *J* = 19.0, 4.5, 3.3)	7.59 (1H, s)	6.63 (1H, s)
5		2.49 (1H, ddd, *J* = 18.0, 10.7, 1.2 Hz, H_ax_-5) 2.53 (1H, ddd, *J* = 18.0, 6.2, 1.2 Hz, H_eq_-5)	2.80–2.82 (1H, m)		3.15 (1H, dd, *J* =16.0, 4.2, H-eq) 2.92 (1H, dt, *J* = 16.0, 12.3, H-ax)
6	5.94 (1H, s)	4.83 (1H, dd, *J* = 10.7, 6.2)			3.34 (1H, td, *J* = 12.6, 4.2)
8			1.24 (3H, s)		
9	7.53 (1H, d, *J* = 8.8)	2.45 (2H, t, *J* = 7.8)	2.49–2.52 (1H, m) 3.03 (1H, dd, *J* = 18.0, 3.2)	6.65 (1H dq, *J* = 15.6, 1.8)	
10		1.61 (2H, sextet, *J* = 7.8)		7.13 (1H, dd, *J* = 15.6, 6.8)	
11	2.60 (3H, s)	0.92 (3H, t, *J* = 7.8)	2.44–2.47 (2H, m)	2.05 (3H, dd, *J* = 6.8, 1.8)	
12	1.48 (3H, s)	1.38 (3H, s)	1.55–1.60 (2H, m)	1.85 (3H, s)	1.47 (3H, s)
13	3.72 (1H, d-like, *J* = 17.0), 3.77 (1H, d-like, *J* = 17.0)		1.20–1.35 (2H, m)	8.04 (1H, dd, *J* = 7.8)	3.72 (3H, d, *J* = 12.6)
14			1.20–1.35 (2H, m)	7.70 (1H, t, *J* = 7.8)	
15	2.72 (1H, d, *J* = 16.2), 2.75 (1H, d, *J* = 16.2)	2.09 (3H, s)	1.20–1.35 (2H, m)	7.90 (1H, dd, *J* = 7.8, 0.6)	2.65/3.03 (each 1H, dt, *J* = 18.0, 7.2)
16	4.25 (1H, m)		1.20–1.35 (2H, m)		1.64 (2H, pentet, *J* = 7.2)
17	1.28 (3H, t, *J* = 6.4)		0.90 (3H, t, *J* = 7.2)		1.30–1.33 (2H, m)
18					1.30–1.33 (2H, m)
19					1.30–1.33 (2H, m)
20					1.30–1.33 (2H, m)
21					0.91 (3H, t, *J* =7.2)
OH-3					5.42 (1H, br s)
OCH_3–_16	3.21 (3H, s)				
OH-4	13.4 (1H, s)				
OH-8	3.50 (1H, br s)/4.15 (1H, br s)				
OH-8b	4.15 (1H, br s)/3.50 (1H, br s)				

**Table 2:** ^13^C-NMR data for compounds **2**–**5** (*δ* in ppm, 150 MHz for ^13^C NMR in CDCl_3_).

No.	1	2	3	4	5
1	108.8	12.3	67.2	149.8	130.4
2	134.0				130.0
2a					22.0
2b					14.2
3	114.8	205.4	170.9	161.8	159.0
3a			144.5		
4	161.2	48.8	25.9	114.0	115.8
4a	110.2	146.8		143.5	140.9
4b	125.3				
5	149.2	37.9	40.7	126.7	30.2
6	123.5	67.9	63.2	151.0	43.1
7	198.4	85.1	198.5	84.9	84.2
7a			148.9		
8	79.8	195.6	19.2	192.8	192.1
8a	157.3	132.0		122.7	124.7
8b	97.2				
9	122.7	45.0	41.8	131.8	
10	203.1	17.2	209.1	137.2	
11	26.6	13.6	43.4	18.8	
12	23.0	16.2	23.5	27.3	17.4
13	48.9		29.0	129.1	54.9
13a			29.0		170.9
14	206.1	170.2	29.0	132.3	203.9
15	50.3	21.3	31.4	127.8	42.8
16	73.4		22.4	125.8	23.5
17	23.0		13.9	168.5	29.1
18					29.1
19					31.7
20					22.8
21					13.9

**Table 3:** Antifungal activity of five sufficient compounds isolated from the culture broth of *A*. *punica* 04107M (diameter of the zone of growth-inhibitory fungicidal zone is given in mm, including the diameter of the disk, which is 8 mm).

Test Microorganism	Isolated Compounds					
	1	2	3	4	5	Ketoconazole
*A*. *niger*	15.4 ± 0.7	29.1 ± 3.5	29.3 ± 1.9	32.0 ± 1.8	27.5 ± 2.8	34.2 ± 1.8
*P*. *italicum*	17.8 ± 1.2	28.5 ± 2.1	29.4 ± 1.4	28.3 ± 3.1	17.5 ± 2.2	35.9 ± 2.3
*C*. *albicans*	16.2 ± 5.4	27.6 ± 3.9	36.2 ± 3.6	31.2 ± 3.5	28.0 ± 3.1	39.3 ± 3.1
*S*. *cerevisiae*	12.9 ± 1.1	30.1 ± 4.0	21.9 ± 2.5	28.2 ± 2.8	27.3 ± 1.4	34.2 ± 1.1

Inhibitory zone diameter (mm); ± inhibitory zone; positive control (STD): ketoconazole. Each value represents the mean ± SD.

**Table 4:** MIC values of compounds **2**–**5** in μg/mL against four fungi strains.

Compounds	*A*. *niger*	*P*. *italicum*	*C*. *albicans*	*S*. *cerevisiae*
**2**	>100	>100	>100	43.45 ± 2.33 ^a^
**3**	>100	>100	32.87 ± 2.19 ^a^	>100
**4**	29.65 ± 3.54 ^a^	>100	58.43 ± 1.51 ^a^	>100
**5**	>100	>100	>100	>100
Ketoconazole	4.10 ± 0.84 ^a^	5.34 ± 2.56 ^a^	10.88 ± 5.67 ^a^	3.57 ± 0.98 ^a^

^a^ Each value represents the mean ± SD.
